# Acetolysis modifications to process small pollen samples swabbed from live bees

**DOI:** 10.1093/jisesa/iead098

**Published:** 2023-11-06

**Authors:** Kirsten Warcup, Bethany Roberton, Katherine Kral-O’Brien, Jason Harmon

**Affiliations:** Department of Biological Sciences, North Dakota State University, NDSU Dept. 2715, PO Box 6050, Fargo, ND 58108-6050, USA; School of Natural Resource Sciences, North Dakota State University, NDSU Dept. 7650, PO Box 6050, Fargo, ND 58108-6050, USA; School of Natural Resource Sciences, North Dakota State University, NDSU Dept. 7650, PO Box 6050, Fargo, ND 58108-6050, USA; School of Natural Resource Sciences, North Dakota State University, NDSU Dept. 7650, PO Box 6050, Fargo, ND 58108-6050, USA

**Keywords:** floral resource, pollen grain, pollinator, nonlethal, sampling

## Abstract

Understanding the resources bees use is essential because we depend greatly on their ecosystem services, and this information could help guide conservation efforts. One way to identify the flowers that bees visit is to collect pollen directly from the bee and then identify the pollen with plant taxa. However, the current method for processing such pollen samples, acetolysis, is designed for samples such as those collected across individuals (e.g., pollen trap), bee nests, or, at the very least, from pollen pellets collected from live bees or from the exhaustive removal of pollen from lethally collected individuals. Smaller samples, including those down to just a few pollen grains sampled from live bees, could facilitate additional opportunities for bee-pollen research, if they can be processed effectively. We present a revised acetolysis methodology designed specifically for processing small pollen samples, so that they can then be used for more accurate identification. Using pollen samples from cotton swabs directly applied to live bees in the field, we demonstrate the effectiveness of our methodology for processing small pollen samples, including samples too small to be visually detected. This methodology can permit nonlethal collections in the field from a greater number of bee species.

## Introduction

Pollen is an important and protein-rich component of a bee’s diet (Nicolson 2011, Huang 2012). Thus, understanding the pollen bees collect can contribute to land management decisions that support bee conservation. To accurately identify this pollen, it needs to be collected and then processed using methods such as acetolysis. Acetolysis is a palynological technique used to process pollen and prepare it for morphological identification via light microscopy. The reaction between the pollen and the acetolysis mixture, a 9:1 ratio of acetic anhydride to sulfuric acid, removes impurities from the outer pollen wall, so identifying characteristics can be viewed (Jones 2014). Acetolysis is a favorable method for pollen processing because it is inexpensive and does not require specialized equipment. However, acetolysis is not typically used with smaller pollen samples, including those down to just a few pollen grains collected from an individual bee in the field, because current protocols have a high risk of pollen loss. Therefore, an accessible way to process pollen for morphological identification does not yet exist for small pollen samples.

Developing a methodology that can process smaller pollen samples has the potential to facilitate additional research approaches for a wider range of bee species and nonlethal methods. Current methods for collecting larger pollen samples often rely on nest traps from social bees or using individuals from bee species that store pollen in corbicula, or pollen baskets (Dimou et al. 2006, Hoover and Ovinge 2018, Minahan and Brunet 2018). However, without using lethal collections, it can be difficult to collect sufficient pollen from numerous other species, especially solitary bees. The unnecessary removal of individuals from a population is becoming generally discouraged (Boyle et al. 2018, Tepedino and Portman 2021). Therefore, exploring alternative methods of nonlethal pollen collections that could be used in place of lethal collections is essential. However, without a way to analyze small pollen samples, it is difficult to answer research questions with individuals across bee species.

In the present study, we sought to (i) revise an acetolysis methodology to increase its effectiveness for use on small pollen samples collected nonlethally from bees in the field and (ii) use the revised acetolysis methodology to assess whether quick swabbing of live bees can yield a pollen sample that can undergo further analysis.

## Methods

### Pollen Collection

**Fig. 1.**
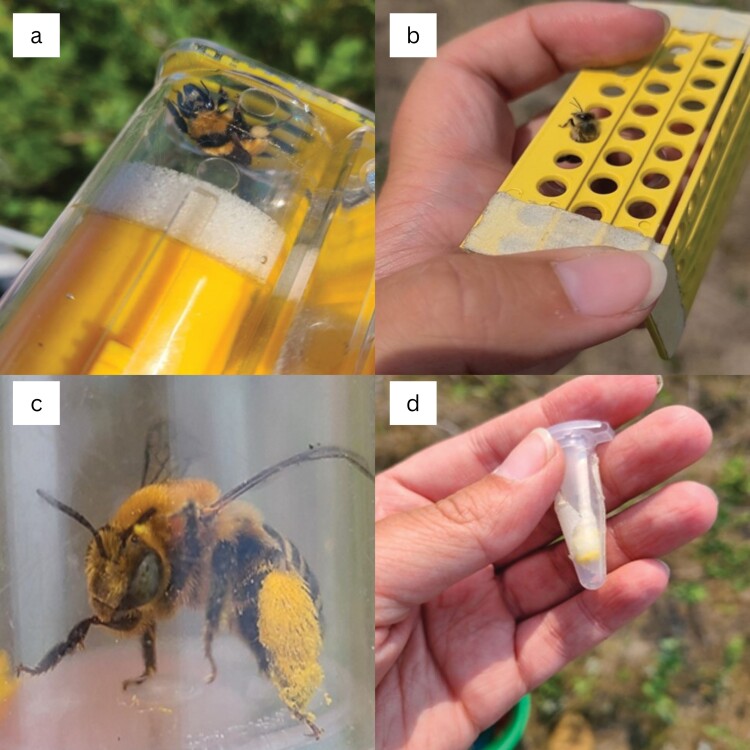
a) A bumble bee in a queen cage before being swabbed with a cotton swab, b) a honey bee crawling through a pollen trap and leaving pollen loads behind in jar, c) a bee in a jar with visible pollen loads before being swabbed with a cotton swab, and d) a cotton swab in a microcentrifuge tube that was used to swab a bee (photograph credit: Bethany Roberton).

We collected pollen samples from bees using nonlethal methods at the Central Grasslands Research Extension Center near Streeter, North Dakota (46°45ʹN, 99°28ʹW). We conducted floral visitor surveys along 96, 100-m transects and caught all bees visiting the reproductive part of flowers within 1 m on each side. After netting bees, we collected pollen from each individual with a cotton swab in slightly different ways depending on the bee body size to optimize collection. Most bumble bees were briefly held in a honeybee queen cage (Fig. 1a), and we used a swab to remove pollen pellets through the cage slots. However, some bumble bees, such as *Bombus borealis*, were small and quickly escaped the honeybee queen cage. We swabbed these individuals while they were inside a jar. Additionally, we found that placing bumble bees into clear plastic bags was also effective at collecting pollen pellets because it allowed for more manipulation without causing as much stress as the honeybee queen cage. Honey bees were allowed to walk through a pollen trap because it efficiently removed the pollen pellets into a jar to be collected (Fig. 1b). If either the bumble bees or honey bees did not have pollen in their corbiculae, they were still swabbed and noted as not having visible pollen on them. Finally, all other bees were held in a jar and directly swabbed with a cotton swab (Fig. 1c and d). We specifically swabbed the hind legs and underneath the abdomen of each bee where pollen is stored for most species during foraging. We targeted these areas to get as much pollen as we could for our sample. To minimize any contamination of pollen samples, we used a new cotton swab for each bee and wiped each cage, pollen trap, and jar with distilled water between uses. We placed all pollen samples in a 1.5-ml microcentrifuge tube and left them to dry in a container with silica gel beads (Dinato et al. 2020) for 24–48 h to prevent ice crystal formation upon freezing and preserve the morphology of as many pollen grains as possible. After drying, the samples were stored at −17 °C. We collected additional data on each bee in the field, including a photo of the bee for identification (except for honeybees), the species and height of the flowering plant the bee was visiting, and whether pollen was visible on the bee before the cotton swab sample was collected.

### Pollen Processing

To process the pollen samples, we performed an acetolysis method. Acetolysis involves a reaction between acetic anhydride and sulfuric acid. The reaction removes impurities, lipids, and other unwanted substances from the grains, revealing the topography and enhancing important morphological characteristics of the grains (Jones 2014). After acetolysis, pollen grains are stained and then observed under a light microscope for identification. In our study, we followed the acetolysis procedure outlined in Jones (2014) and made modifications to optimize the protocol for extremely small pollen samples (Table 1).

**Table 1.** A table outlining the main changes made to the protocol by Jones (2014) to optimize the protocol for tiny pollen samples on cotton swabs

Parameter	Our protocol	Jones’ (2014) protocol
Sample starting material	Pollen on a cotton swab	Pollen in water, in other liquids, in or on pollinators, on cellophane tape, on filter paper, and pollen pellets
Pollen sample size	Tiny	Small to very large
Number of water rinses	1	3 or more
Ethanol rinse (yes/no)	No	Yes
Volume of acetolysis mixture needed per sample	1 ml	1–5 ml
Water bath duration	10 min	5–25 min
Straining	Not necessary	Necessary if pollinator body parts remain following the water bath step

A list of materials needed for acetolysis is below:

▪ PPE
  ◦ Lab coat
  ◦ Face shield
  ◦ Goggles
  ◦ Nitrile gloves
▪ Chemicals
  ◦ Concentrated sulfuric acid
  ◦ Acetic anhydride
  ◦ Glacial acetic acid
  ◦ 95% ETOH
  ◦ Safranin O powder
  ◦ Glycerol
▪ Lab equipment and consumables
  ◦ Microcentrifuge
  ◦ Heat block
  ◦ One thousand-liter pipette and tips
  ◦ Wooden stir sticks
  ◦ Microcentrifuge tubes
  ◦ Microscope slides and cover slips

**Fig. 2.**
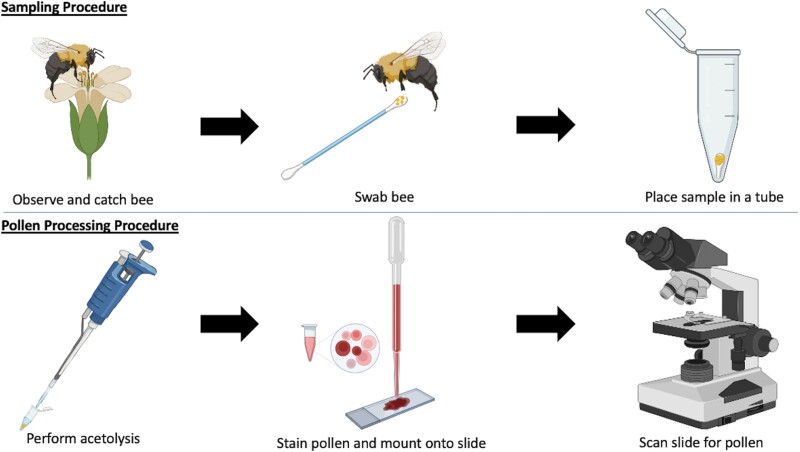
A flowchart outlining the main steps in the pollen sampling and processing workflow. Pollen samples were collected nonlethally from bees on cotton swabs and were frozen at −17 °C. Acetolysis was used to process the pollen samples and prepare them for identification with a light microscope (Created with BioRender.com).

Our full procedure is outlined in Fig. 2.

**Fig. 3.**
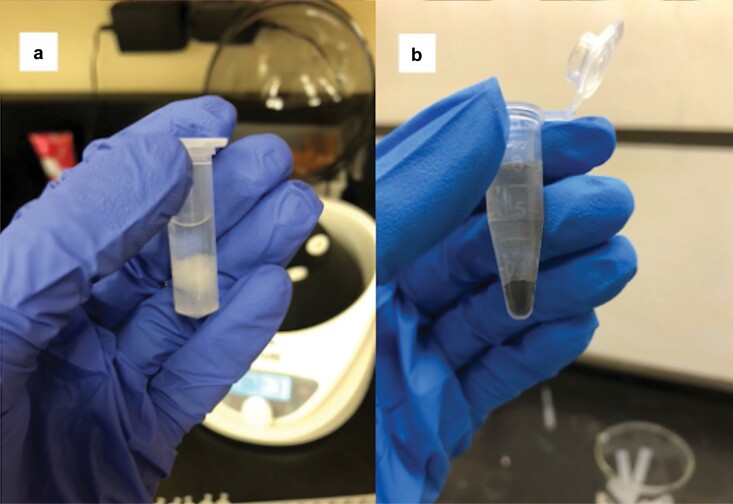
a) The first step of the modified acetolysis procedure where the cotton has been removed from the cotton swab and placed in glacial acetic acid and b) black pollen grains visible at the water rinse step of acetolysis (photograph credit: Kirsten Warcup).

To prepare samples for acetolysis, we removed the cotton containing the pollen sample from the end of the cotton swab and placed it in a microcentrifuge tube (Fig. 3a). We added 1 ml of glacial acetic acid to each sample and centrifuged the samples for 3 min at 1,060 × g. We prepared the acetolysis mixture by combining acetic anhydride and sulfuric acid in a 9:1 ratio. We decanted the glacial acetic acid from the tubes and added 1 ml of the acetolysis mixture to each tube. We stirred each tube with a clean wooden stir stick, pushed the cotton to the bottom of the tube, and added the samples to a preheated water bath (100 °C) for 10 min. We observed that samples containing a large amount of pollen commonly underwent a dramatic color change during the heating process; however, we did not collect data on this process. After the water bath, the cotton was completely dissolved leaving only the pollen sample that was on the cotton. The samples were cooled and then centrifuged at 1060 × g for 3 min. We aspirated the acetolysis mixture with a pipette and added 1 ml of glacial acetic acid to each sample. We centrifuged the samples for 3 min at 1,060 × g and the glacial acetic acid was aspirated. We performed a water rinse by taking a squirt bottle containing distilled water and forcefully squirting water into the tube until it was three-fourths full. Once the samples all had water, they were centrifuged at 1,060 × g for 3 min, and subsequently, the supernatant was aspirated. In some samples, the pollen could be visualized in the form of small black dots at this step (Fig. 3b). We aspirated the water, leaving the pollen ready for staining.

### Pollen Staining and Microscopy

A glycerol solution (Danti et al. 2011) and Safranin O stain (Green 1991 [as cited in Jones 2012]) were used to stain the pollen and prepare it for microscopy. To make the glycerin solution, we mixed 25 ml of glycerin with 25 ml of distilled water until combined. We prepared the Safranin O stain by adding 1 gram of Safranin O powder to 100 ml of 50% ethanol and mixing for 3 min. Both solutions can be made ahead of time and stored at room temperature. To stain the pollen, we added one drop of the glycerol solution and one drop of the stain to each microcentrifuge tube following the water rinse, mixed the samples by flicking the tube, and briefly centrifuged the tubes to collect all the liquid and pollen at the bottom. Using a new disposable pipette for each tube, we pipetted all the liquid and pollen from the tube and deposited it onto two clean microscope slides. We carefully placed cover slides on each slide to ensure no air bubbles formed, sealed the slides with clear nail polish, and allowed them to dry before being scanned for pollen.

### Slide Scanning

Slides were initially scanned for pollen. To complete this, we placed the slide on a light microscope and viewed it at 4x magnification. Starting at the top, we scanned the slide from left to right and continued this process until the entire slide had been viewed. If we came across something that looked like pollen, we increased the magnification to 10× or 40× to confirm that we were viewing pollen. If any pollen was visible, we marked the slide for further analysis, and if no pollen was present, the slide did not undergo further analysis.

## Results

**Fig. 4.**
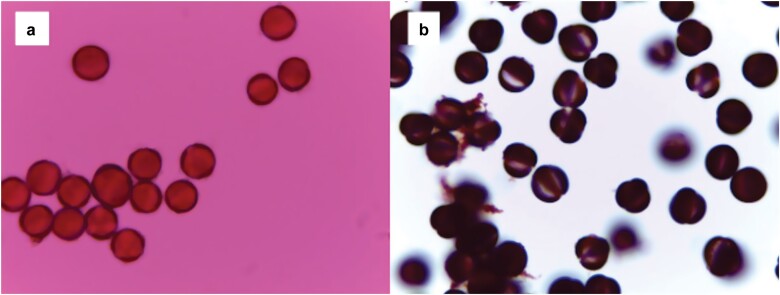
a) A microscope slide of pollen grains before acetolysis using a large amount of pollen collected from honeybee hives for practice, b) and a slide of pollen grains following acetolysis using pollen from the same practice source. The polarity of the pollen grains and the apertures are more easily viewed in the second photo (photograph credit: Kirsten Warcup).

**Fig. 5.**
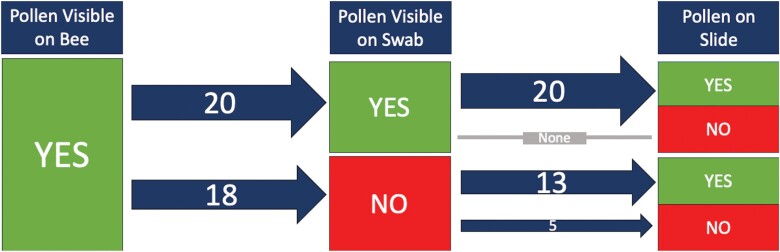
A flowchart following the presence of pollen on a microscope slide if it was visible on the bee and then if it was visible on the cotton swab. The numbers on the arrows indicate the number of samples, and the arrow thickness was visually adjusted to represent the number of samples processed.

**Fig. 6.**
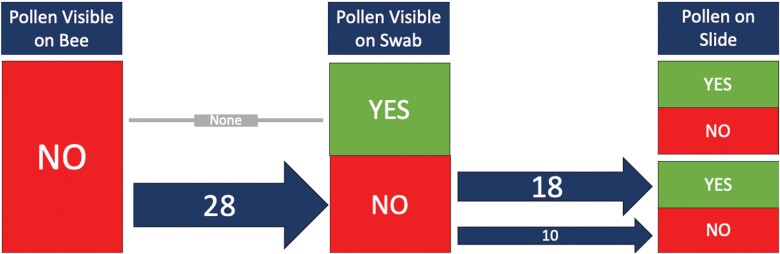
A flowchart following the presence of pollen on a microscope slide if it was not visible on the bee and then if it was visible on the cotton swab. The numbers on the arrows indicate the number of samples, and the arrow thickness was visually adjusted to represent the number of samples processed.

In the summer of 2021, we swabbed pollen samples from 112 bees across four families (6 Andrenidae, 80 Apidae, 18 Halictidae, and 8 Megachilidae). We were able to successfully process our samples and mount them on microscope slides to be used for plant taxa identification (Fig. 4). When examining the consistency of finding pollen grains on our samples taken directly from bees caught in the field, we found that pollen was often on the cotton swab if pollen had been visible on the bee (53%, *n* = 38) (Fig. 5). In some cases, there was pollen visible on the bee, but pollen was not detected after processing (13%, *n* = 38) (Fig. 5). This suggests that either the pollen was not captured in the swabbing process, or the pollen was lost during processing. Interestingly, even if pollen was not visible on the bee or on the swab, we sometimes had a few pollen grains on the slide following acetolysis (64%, *n* = 28) (Fig. 6). This suggests that either the pollen was an inconspicuous color (such as tan), which made it difficult to see on the bee and the swab, or the pollen sample was so small that it could not be visualized.

## Discussion

Overall, we found that our modified acetolysis method was able to process small amounts of pollen, including samples that were not visible to the naked eye. This finding supports the ability of researchers to collect and process pollen samples for identification from live bees during field surveys or experiments. Our modifications to the acetolysis procedure allowed for samples to be collected on cotton swabs and for seemingly invisible amounts of pollen to be processed. As expected, we found that visible pollen on the bee in the field or on the cotton swab was a strong indicator that pollen would be present on the sample after acetolysis. However, we even observed pollen after processing that was not visible in the field. These results further indicate that quick, nonlethal collections can yield pollen samples.

Although our findings suggest that small pollen samples can be collected and processed through nonlethal collection methods and modified acetolysis, some considerations are necessary for future studies when using this process. First, identifying the pollen grains as plant taxa after acetolysis can still be difficult. Pollen identification via light microscopy requires a well-trained eye and a reference guide specific to the location of pollen collections. Second, sampling bees in this way likely collects only a subsample of the pollen that the bees have collected. Future studies could provide more insights into the effectiveness of swabbing by determining either how much of a pollen load is collected from a bee or if multiple swabs from the same bee produce comparable pollen samples after processing and identification.

Analyzing smaller pollen samples (i.e., even several pollen grains) could provide a wealth of potential information. Just a small number of pollen grains can be sufficient to accurately measure the pollen’s quantitative features (Wrońska-Pilarek et al. 2015), suggesting that small pollen samples, such as the samples collected by swabbing bees, can provide useful morphological descriptions of pollen grains. Furthermore, the ability to process and analyze small samples allows researchers to ask pollen-related questions of several native bee species that carry pollen on the outside of their bodies rather than just a select few. Catching actively foraging bees in the field and processing such small pollen samples also opens up more opportunities to tie pollen collections from individual bees with specific observations of bee behaviors and activity, including within specific experimental treatments in the field. Moreover, optimizing small pollen sample analysis will enable nonlethal collection methods to be used when directly sampling from individual bees.

In summary, our modified acetolysis technique provides an option for researchers to collect pollen nonlethally from a variety of species. Research such as this is important for the conservation of areas that bees use for resources and supports the expansion of using nonlethal methods for collecting information from bees to further aid in their nutrition and conservation management.
